# The HIV Reservoir in Monocytes and Macrophages

**DOI:** 10.3389/fimmu.2019.01435

**Published:** 2019-06-26

**Authors:** Michelle E. Wong, Anthony Jaworowski, Anna C. Hearps

**Affiliations:** ^1^Central Clinical School, Monash University, Melbourne, VIC, Australia; ^2^Life Sciences Discipline, Burnet Institute, Melbourne, VIC, Australia; ^3^Chronic Inflammatory and Infectious Diseases Program, School of Health and Biomedical Sciences, Bundoora, VIC, Australia; ^4^Department of Infectious Diseases, Monash University, Melbourne, VIC, Australia

**Keywords:** HIV, monocytes/macrophages, reservoir, DNAscope, animal models

## Abstract

In people living with HIV (PLWH) who are failing or unable to access combination antiretroviral therapy (cART), monocytes and macrophages are important drivers of pathogenesis and progression to AIDS. The relevance of the monocyte/macrophage reservoir in PLWH receiving cART is debatable as *in vivo* evidence for infected cells is limited and suggests the reservoir is small. Macrophages were assumed to have a moderate life span and lack self-renewing potential, but recent discoveries challenge this dogma and suggest a potentially important role of these cells as long-lived HIV reservoirs. This, combined with new HIV infection animal models, has led to a resurgence of interest in monocyte/macrophage reservoirs. Infection of non-human primates with myeloid-tropic SIV implicates monocyte/macrophage activation and infection in the brain with neurocognitive disorders, and infection of myeloid-only humanized mouse models are consistent with the potential of the monocyte/macrophage reservoir to sustain infection and be a source of rebound viremia following cART cessation. An increased resistance to HIV-induced cytopathic effects and a reduced susceptibility to some antiretroviral drugs implies macrophages may be relevant to residual replication under cART and to rebound viremia. With a reappraisal of monocyte circulation dynamics, and the development of techniques to differentiate between self-renewing tissue-resident, and monocyte-derived macrophages in different tissues, a new framework exists to contextualize and evaluate the significance and relevance of the monocyte/macrophage HIV reservoir. In this review, we discuss recent developments in monocyte and macrophage biology and appraise current and emerging techniques to quantify the reservoir. We discuss how this knowledge influences our evaluation of the myeloid HIV reservoir, the implications for HIV pathogenesis in both viremic and virologically-suppressed PLWH and the need to address the myeloid reservoir in future treatment and cure strategies.

## Introduction

Whilst CD4+ T cells are the primary targets of HIV, myeloid cells also express the HIV primary receptor CD4 and the chemokine co-receptor CCR5, and are also infected *in vivo* by R5-tropic and dual tropic strains of HIV. Monocytes and macrophages are significant mediators of inflammation, and dysregulation of their inflammatory functions either by direct or bystander mechanisms during HIV infection is a key driver of comorbidities with an inflammatory etiology in PLWH. The significance of macrophage infection in viremic individuals is well established: HIV species within individuals become increasingly macrophage-tropic with disease progression (1) and by late stage infection, CD4 T cells are depleted and infected macrophages are a principal reservoir driving viremia (2, 3). Moreover, monocyte and macrophage infection is linked to HIV pathologies including the development of HIV-associated dementia (HAD) by promoting inflammation and production of neurotoxins, and by impaired immunoprotective functions leading to thriving opportunistic infections (4). Currently, the role and relevance of monocytes and macrophages during virologically-suppressed HIV infection remains poorly defined, and the persistence, extent and relevance of a monocyte/macrophage HIV reservoir is not clearly understood. With effective cART, the extent of monocyte/macrophage activation and dysfunction is substantially reduced as compared to untreated PLWH, but is not completely ameliorated (5, 6) and contributes to comorbidities including milder HIV-associated neurocognitive disorders (HAND) (7), cardiovascular disease (8, 9), early immune aging (10, 11) and also all-cause mortality (12) [reviewed by (13)]. In this context, the relative contributions of direct infection of monocytes/macrophages vs. bystander effects of persistent, chronic inflammation remain unclear, but the low frequency of monocyte/macrophage infection, particularly during cART, implies the latter is more relevant. However, the contribution of HIV infected monocytes/macrophages to comorbid disease development and the persistence of the HIV reservoir in the setting of long-term, effective virologic suppression is not well understood and needs to be addressed. In current scenarios of controlled HIV infection with successful cART, many questions remain including the extent to which monocyte/macrophage reservoirs persist, how long lived are HIV-infected macrophages, does it include latently infected cells, is it an important source of cryptic viremia in sanctuary tissue sites such as the brain and other tissues and can it contribute to rebound viremia following cART cessation? These questions will need to be addressed to inform research into HIV cure strategies. This review will focus on the detection and measurement of the monocyte/macrophage reservoir and recent advancements in the field of monocyte and macrophage ontogeny and circulation dynamics which affect the way in which the myeloid reservoir should be evaluated.

## Monocyte/Macrophage Biology

A basic understanding of the origins and functions of monocytes and macrophages forms the foundation for understanding and targeting the myeloid HIV reservoir. Recent discoveries have challenged early dogma that macrophage populations are terminally differentiated cells, sustained through continual replenishment by bone-marrow derived monocytes. Long-lived tissue resident macrophage populations, which are derived from yolk sac-progenitors and fetal liver-derived monocytes, have been described and shown to be capable of self-renewal, independently of circulating monocytes (14). The discovery of this new macrophage niche represents a paradigm shift in the field of macrophage ontogeny, which needs to be reflected in how monocytes and macrophages are evaluated in the context of HIV infection.

### Monocyte Subtypes and Circulation Dynamics

**Figure 1:**
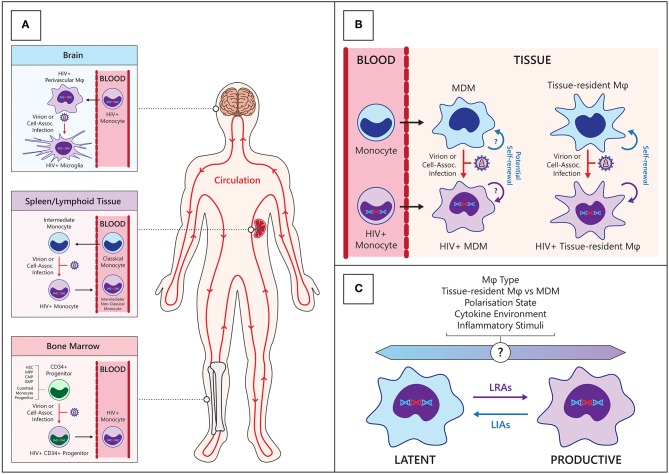
Establishment and maintenance of the HIV myeloid reservoir. **(A)** CD34+ progenitors—hematopoietic stem cells (HSC), multipotent progenitor (MPP), committed myeloid progenitors (CMP), granulo-monocyte progenitors (GMP), and committed monocyte progenitors—in the bone marrow may become infected with HIV then migrate and differentiate into monocytes in circulation (Bottom panel). Classical monocytes differentiate into intermediate monocytes and migrate into tissues such as the spleen, where they may become infected with HIV and re-enter the circulation (center panel). Circulating HIV-infected monocytes can enter anatomical sanctuary sites such as the brain, differentiate into macrophages (ϕ) and thus seed tissue reservoirs (Top panel). **(B)** HIV macrophage reservoirs in tissues can be maintained by infiltrating infected monocytes, de novo infection of monocyte-derived macrophages (MDM) within tissues, and by homeostatic self-renewal of infected tissue-resident macrophages. **(C)** Various endogenous and exogenous factors may influence the state of HIV infection within macrophages. LRAs, latency reversing agents; LIAs, latency inducing agents.

Monocytes are derived from granulocyte/monocyte progenitors in the bone marrow and enter circulation under the influence of the chemokine CCL2 via the CCR2 receptor (15). The monocyte lineage is derived from pluripotent hematopoietic stem cells which progressively differentiate into common CD34+ myeloid progenitors, granulo-monocyte progenitors and committed monocyte progenitors within the bone marrow (16). These cells express CD4 and the coreceptor CCR5, albeit at very low levels, and there is inconsistent data regarding their susceptibility to HIV infection *in vitro* (17–20). Further *in vivo* evidence suggests that a limited CD34+ myeloid progenitor HIV reservoir exists in some individuals (21, 22), although this has not been found in other studies (23, 24). Importantly, the bone marrow is a secondary lymphoid organ to which T cell homing is increased in PLWH (25), and can thus be a site of infection of CD34+ progenitors and monocytes. Following differentiation, these infected CD34+ cells may be involved in trafficking of virus to tissue compartments including the brain (see Figure 1), but the extent to which this occurs *in vivo* is not known. The clinical relevance of the HIV-infected CD34+ progenitor cell reservoir is difficult to assess as there is very limited information regarding its prevalence and persistence in HIV+ individuals on current cART regimens with long term virological suppression.

In circulation, monocytes circulate through blood and lymph with a half-life of ~71 h (26) before migrating into tissues and differentiating into macrophages. Under inflammatory conditions, monocyte turnover is increased and specific monocyte-derived macrophages (MDM) populations are expanded at inflammatory sites. Human monocytes are subdivided into 3 subsets based on surface expression of CD14 and CD16: classical (CD14++/CD16-), pro-inflammatory intermediate (CD14++/CD16+) and “patrolling” non-classical monocytes (CD14+/CD16++) monocytes, which each represent about 90, 5, and 5% of total circulating monocytes, respectively, in healthy individuals (27). Classical monocytes appear to be the first subset to appear in peripheral blood, followed by intermediate and non-classical monocytes (28), with evidence suggesting that populations transition sequentially from classical monocytes to non-classical monocytes via intermediate monocytes (29). To understand how reservoirs are established in different monocyte subsets, a knowledge of monocyte ontogeny, monocyte subset dynamics and migration behavior is required.

Recent modeling of human monocyte circulation dynamics by Tak et al. suggests <10% of classical monocytes mature into circulating intermediate monocytes, and 82–89% of these subsequently mature into circulating non-classical monocytes (30). Interestingly, their data indicated intermediate monocytes spend an average of 1.6 days outside circulation before re-entering circulation as non-classical monocytes, suggesting intermediate monocytes may be infected with HIV in tissues during this trafficking. Discrete pools of non-circulating, mature monocytes have been described in bone marrow (31), spleen (32), and patrolling blood vessel adherent monocytes (33), which are distinct from MDM in tissues (30). This has implications for where monocytes/macrophages can be infected with HIV and how they may subsequently traffic virus to other regions in the body (Figure 1).

The trafficking of individual monocyte subsets is influenced by their distinct chemokine receptor expression profiles and has implications for their ability to seed tissue reservoirs. Classical monocytes express high levels of CCR2 and migrate out of the bone marrow and into sites of infection and inflammation in response to CCL2, whereas non-classical monocytes express very low levels of CCR2 and high levels of the fractalkine receptor CX3CR1, allowing blood-vessel wall patrolling functions (34–36). Intermediate monocytes express intermediate levels of CCR2 (37) and are efficiently recruited to lymph nodes following immune activation/inflammation (38). It has been reported that the more mature CD16+ monocytes are preferentially infected with HIV (39), which is consistent with their higher level of CCR5 expression as compared to classical monocytes. This observation is hard to reconcile with a model in which the HIV reservoir in monocytes is established in precursor cells in the bone marrow and HIV-infected monocytes progress through their maturation pathway in peripheral circulation. The preferential infection of CD16+ subsets may also be due to their heightened ability to undergo extravasation from blood into tissues such as lymph nodes, where they are more likely to encounter productively infected cells. This has clinical implications, as HIV+ intermediate monocytes preferentially migrate across the blood brain barrier and contribute to HAND (40). This represents a potential mechanism for trafficking of virus between tissue reservoirs during HIV infection, especially as the intermediate monocyte subset is expanded in inflammatory states including viremic HIV infection (41, 42).

### Macrophage Polarization and Heterogeneity

Given their range of functions in tissue homeostasis and protection against infection, macrophages are highly sensitive to changes in the local cytokine environment and exhibit extensive heterogeneity [recently reviewed in (43)]. In response to environmental stimuli, macrophages can be primed toward more pro-inflammatory, anti-inflammatory or tissue remodeling responses. Macrophages can be polarized *in vitro* to inflammatory M1 and anti-inflammatory M2 macrophages using a range of cytokines and maturation factors (44). While the *in vivo* relevance of these experimentally induced states has been debated, M1 and M2 macrophages may be considered as representing extremes of macrophage polarization, with macrophage populations *in vivo* either falling on a spectrum between them or exhibiting overlapping M1-like and M2-like phenotypes (45). *In vivo*, splenic macrophages can be polarized (46) and display similar phenotypes to those generated in *in vitro* systems. Thus, macrophage polarization is a useful tool to highlight the different responses which may be elicited in different cytokine environments.

Macrophage polarization is relevant in HIV infection where acute and chronic inflammation, characterized by different macrophage polarization states, are present at different stages of disease (47). Disease progression is associated with a shift in the cytokine environment from a type-1 inflammatory environment to a type-2 immunosuppressive environment (48–50) and is posited to drive the shift from HIV infection-driven M1 polarization of macrophages (51) toward an M2 polarization state (47, 52). The effects of M1 and M2 polarization *in vivo* are varied, with each stimulating pathways that both benefit and inhibit host defenses, as discussed extensively (52). Although the implications for HIV infection and pathogenesis are subject to debate, it is clear that macrophage polarization is an influential factor with respect to establishment of HIV infection and pathogenesis (Figure 1). *In vitro* macrophage infection studies have shown that relative to unpolarized macrophages, both M1 and M2 macrophages have impaired function during chronic and acute HIV infection (53) and are refractory to HIV infection (54–58), albeit through different mechanisms. Extensive work by Graziano et al. who have investigated *in vitro* macrophage plasticity, demonstrates that further stimulation of polarized macrophages with the same or opposing cytokines can modulate HIV restriction (58). This emphasizes the intricate link between flux in the cytokine environment and HIV macrophage reservoir dynamics. Changes in macrophage polarization and functions may therefore act as a mechanism for controlling latency and may contribute to stochastic reactivation and viremic “blips” often seen in patients receiving cART, but this remains to be investigated *in vivo*. Moreover, macrophage polarization can induce differential expression of drug efflux transporters, perhaps contributing to sub therapeutic antiretroviral concentrations (59). Recently, Ganor et al. investigated the polarization states of *ex vivo* urethral macrophages from HIV-, and virologically suppressed HIV+ individuals and reported an intermediate polarization state (Mi) which expresses both M1 (IL-1R) and M2 (CD206) markers. Mi-polarized macrophages are enriched in HIV+ individuals and preferentially infected by HIV (60). Thus, whilst *in vitro* macrophage polarization is a useful and relevant tool to investigate how macrophage heterogeneity and plasticity may influence HIV infection, more *ex vivo* analyses are clearly required to fully understand infection dynamics in this complex cell type.

### Macrophage Self-Renewal

Our understanding of macrophage heterogeneity has expanded with the discovery of self-renewing tissue resident macrophages which originate from embryonic yolk sac and fetal liver precursors rather than from circulating monocytes. Experiments in mice have provided evidence for self-renewing populations of myeloid cells including epidermal Langerhans cells (61) and microglia (62) which were maintained via host-derived local expansion, independently of donor-derived circulating monocytes. Murine fate-mapping (63) and lineage tracing experiments have since revealed 3 waves of macrophages sourced from embryonic yolk sac progenitors, fetal liver progenitors and hematopoietic stem cells [reviewed in (64)]. The extent to which tissue macrophage populations are maintained by tissue resident (TR) macrophage expansion or MDM is unique to different tissues, with microglial and Langerhans cell populations largely self-renewing, while gut macrophage populations are largely monocyte-derived (65, 66). Trauma or inflammation can affect these dynamics with greater monocyte infiltration to bolster local macrophage populations (67); however, multiple studies have shown inflammation-recruited MDM populations to be relatively transient (68, 69). Under homeostatic conditions, macrophage half-lives vary between tissues from <6 days for dermal CD14+ MDM (70) to ~2 months for alveolar macrophages (71), but Réu et al. have also suggested that the average age of microglial cells is 4.2 years whilst individual microglial cells could potentially be decades old (72). Bone-marrow derived macrophages can give rise to long-lived self-renewing heart (73) and lung (74) macrophages in humans, suggesting some MDM have potential to form a stable, self-renewing, tissue resident population in different tissues (Figure 1). To the extent that these cells are infected with HIV, this has significant implications for the maintenance of long-lived macrophage HIV reservoirs comparable in duration to those found in memory T cells, and their persistence in settings of treatment with cART. Moreover, while these MDM and tissue resident macrophages can be induced toward similar functions by local stimuli, they are discrete populations with different responses and functions, even within the same tissue. Currently, it is not known which macrophage populations are preferentially infected by HIV with respect to tissue type, ontogeny, and polarization legacy, and current literature has largely been restricted to whole macrophage populations within different tissues. Given the highly heterogeneous nature of human macrophages, future studies into HIV macrophage reservoirs will need to consider different macrophage populations.

## Techniques to Study the Myeloid HIV Reservoir

Sensitive and specific techniques are needed to measure the scope of the HIV reservoir which persists after cART given the low frequency of infected cells. Current techniques used primarily in T cells to detect HIV infection are subject to limitations [reviewed in (75, 76)] and a combination of techniques will likely be required to comprehensively and specifically map the myeloid HIV reservoir. Detection of HIV-infected cells is often performed using qPCR for HIV DNA, qRT-PCR for HIV RNA, and quantitative viral outgrowth assays (qVOA) using isolated cells. Some HIV DNA qPCR assays are not specific for integrated HIV DNA, or do not distinguish between replication competent and defective proviruses, thus overestimating the amount of latent, activatable viral genomes. Heiner et al. estimate only 5% of proviruses within T cells are intact, and potentially replication competent (77) and there is no information on this proportion in myeloid cells. The qVOA specifically detects replication competent viruses, and quantifies the inducible, replication competent HIV reservoir; however, this technique can underestimate the HIV reservoir as it depends on the production and release of HIV capsid protein (p24) from productively-infected cells and may not detect latently-infected cells. Indeed, Ho et al. estimate that the qVOA measures only 1% of the HIV infected cell population due to inefficient induction of productive infection from quiescent, latently-infected cells (78). It also requires large numbers of cells which can be very difficult to acquire, particularly from tissue samples, and is thus less feasible for use with cells such as macrophages. These qPCR and qVOA HIV detection assays are also indicators of infection in bulk tissue samples and are unable to identify the specific cellular source of HIV. The use of these techniques to detect the HIV monocyte/macrophage reservoir is thus vulnerable to T cell contamination during sample preparation as HIV-infected myeloid cells are a relatively low frequency target in patients receiving cART.

Recently, more specific and sensitive techniques such as DNAScope and RNAScope, and single cell assays, have overcome some technical limitations to studying the monocyte/macrophage reservoir. DNA and RNA Scope are improved *in situ* hybridization technologies which couple highly selective probe sets with extensive amplification, allowing for detection of both HIV DNA and RNA with single cell resolution coupled with immunohistochemistry with appropriate lineage markers to identify infected cells. This technology has been used in tissue sections obtained from animal models of HIV infection (see below) to study the impact of cART on reservoirs in specific cell types using tissues not readily available from human subjects. Moreover, viral DNA and RNA can be detected simultaneously using Scope technology allowing detection of potentially latent HIV DNA+RNA- cells (79). Ko et al. have also used DNAScope and immunohistochemistry to detect HIV DNA in CD68 or CD206 expressing macrophages and microglia, but not in astrocytes, in human brain tissue in the setting of suppressive cART (80). These data demonstrate that this technology may be used to quantify and identify specific cell populations harboring integrated HIV DNA and actively transcribing HIV RNA, or to demonstrate potential latently-infected cells, all in the context of native tissue microenvironments (81). Other single cell techniques including laser capture microscopy to isolate nuclei or whole cells from fixed tissue samples, single cell transcriptomics, and the Full-Length Individual Proviral Sequencing assay, which identifies near full length intact proviral sequences (77), although time-consuming and laborious, complement existing detection assays to offer new insights into specific cells of the HIV reservoir. These methods are especially useful for the study of cells in tissue samples whose behavior with respect to viral production often correlates poorly with peripheral blood compartments (82, 83). Given the low frequency of latently infected cells and the small size of the myeloid reservoir, these emerging techniques which allow for the interrogation of single cells, in context of cellular subtype and tissue microenvironment, address technical weaknesses inherent on older detection technologies and are ideal for contemporary studies measuring the myeloid reservoir.

## Monocyte/Macrophage HIV Reservoirs in HIV+ Individuals

The extent and relevance of the monocyte and macrophage HIV reservoir in humans has been a subject of debate. The drastic reduction in reservoir size with successful cART, limited access to relevant tissue samples and inherent weaknesses in detection techniques has failed to convincingly demonstrate the presence of a replication-competent monocyte/macrophage HIV reservoir in virologically-suppressed individuals. Nevertheless, data showing the presence of HIV DNA in myeloid cells in virologically suppressed individuals, and the persistence of myeloid cell associated comorbidities, argue against the dismissal of this important reservoir.

### Monocytes and Macrophages During Untreated HIV Infection

Macrophage reservoirs are seeded within the first few days (84) and are sustained throughout HIV infection, including during the asymptomatic stage of disease during which it can still drive pathogenesis. Jambo et al. detected HIV in alveolar macrophages present in chronically infected, cART-naïve HIV+ individuals using a fluorescence *in situ* hybridization-based flow cytometry assay, specifically gating on CD206 positive macrophages and excluding CD3+ T cells (85). Detection of an infected macrophage population was associated with impaired phagocytic activity, although this may not be restricted to infected cells as both direct (86, 87) and bystander mechanisms (88) of macrophage phagocytic inhibition have been described. Central nervous system (CNS) infiltration by HIV occurs as early as 8 days post infection (89), and within 1 year, structural changes in the brain are detectable (90). Increased glial cell activation and neuronal injury is observed in HIV infected, cART-naïve individuals (91) and HIV has been detected in microglia in post mortem samples of brain from asymptomatic HIV+ individuals (92). Yakasai et al. detected symptomatic HAND in 40% of cART-naïve HIV+ individuals (93) and brain injury has been associated with HIV DNA in peripheral blood monocytes (94, 95). These data suggest an ongoing contribution of infected monocytes and macrophages to disease pathology over the course of HIV infection, including during asymptomatic, chronic infection (96).

### The Monocyte Reservoir During cART-Mediated Virological Suppression

In individuals receiving cART, the extent of *in vivo* infection of monocytes and macrophages is more contentious, and evidence is limited. Monocytes have host-cell restriction mechanisms which limit HIV infection, including the restriction factors SAMHD1 and APOBEC3, and cellular microRNAs (97–102); however, several groups have detected HIV in blood monocytes in PLWH with defined cART status including virologically suppressed individuals (Table 1) (39, 103–116). In addition to the studies summarized in Table 1, other groups have detected HIV in monocytes of suppressed individuals (117–121) but have not been included in Table 1 due to insufficient information to determine the virological status of participants. Most of the above studies detected HIV in monocytes to varying extents in 30–100% of donors tested; however integrated DNA and replication competence were assessed in only a small number of studies (103–106). Many studies utilized patient study groups with mixed virologic histories and variable treatment effectiveness, and were limited by small sizes, which may explain the variation in results. Moreover, not all studies determined the degree of contamination of monocyte preparations with T cells. This is especially relevant in context of older monocyte isolation techniques which do not achieve the extremely high purity expected of FACS sorting. Nevertheless, studies satisfying the criteria discussed above have detected HIV DNA in monocytes isolated from long-term virologically suppressed individuals, with highly purified monocyte isolates (106–108, 114) (Table 1). PCR-based detection of HIV DNA is highly sensitive; however, HIV DNA was not detected in all donors, and detection rates varied, between studies suggesting the extent of the persisting monocyte reservoir under cART is highly variable and/or very small. Indeed, Spivak et al. were only able to detect HIV DNA in monocytes from 2 of 13 patients on cART (112) and Almodóvar et al. were unable to detect HIV DNA in monocytes from any of the 14 donors with virologic suppression they studied (109). Moreover, while Cattin et al. were able to detect HIV DNA in monocytes isolated from 4 of 10 HIV+ individuals receiving cART, none of these individuals had detectable levels of integrated HIV DNA (116). Difficulties in consistently detecting HIV infection of monocytes has engendered skepticism, resulting in a data set which, while suggestive of the persistence of a reservoir, has yet to demonstrate replication competent virus in monocytes, with solid evidence of no T cell contamination or phagocytosis. The technical advances in detection methodologies described above such as Scope technology will be required to convincingly demonstrate the existence and relevance of the monocyte/macrophage reservoir in the cART era.

**Table 1:** Evidence for the infection and persistence of HIV within monocytes.

**Reference**	***n*=**	**Isolation technique (purity)**	**Virological suppression**	**Years on cART**	**Detection methodology and number positive**	**Replication competence**	**T cell specific contamination**
Lambotte et al. (103)	5	Microbead (>96% purity)	Yes for all	1–2	HIV DNA detected by PCR. 5/5	N.D.	<2% CD3+ cells detected
					HIV RNA detected by PCR. 5/5	HIV RNA detected in supernatant	Not specifically assessed
Calcaterra et al. (104)	11	Plate adherence and complement-induced CD3 lysis (>94% purity)	*n* = 7 Yes *n* = 4 No	1–3	Total and integrated HIV DNA detected by PCR. Total: 9/11, Integrated: 3/11	N.D.	Not detected
					Soluble HIV p24: 0/11	Not detected	N.D.
Sonza et al. (105)	10	Plate adherence ± microbead CD3 depletion (purity not determined)	Yes for all	N/A	Integrated HIV DNA and 2LTR circles detected by PCR. Integrated: 10/10, 2LTR: 4/5	N.D.	≤0.1% contain TCR mRNA
					Multiply spliced (ms) and virion associated RNA detected by PCR. msRNA: 4/5, Virus: 5/10	HIV RNA detected (supernatant and cell lysates)	
Zhu et al. (106)	7	Microbead and FACS sorting (98.3–100% purity)	Yes for all	2–4	HIV DNA and RNA detected by PCR. DNA:7/7, RNA: 7/7	HIV msRNA detected	N.D.
Garbuglia et al. (107)	18	Microbead (>99% purity)	Yes for all	>2	HIV DNA detected by modified HIV-1 Amplicor assay. 16/18	N.D.	N.D.
Delobel et al. (108)	3	Microbead and FACS sorting (>99% purity)	Yes for all	7–12	HIV DNA detected by PCR. 3/3	N.D.	N.D.
Almodóvar et al. (109)	14	Microbead (95–99% purity)	*n* = 12 Yes *n* = 2 No	N/A	HIV DNA detected by PCR. 0/14	N.D.	≤1.1% CD3+ cells detected
Ellery et al. (39)	17	Microbead and plate adherence, or FACS sorting (>95% purity)	*n* = 5 Yes *n* = 12 No	N/A	HIV DNA detected by PCR. 16/17	N.D.	≤0.01% contained TCR mRNA
Gibellini et al. (110)	34	Microbead(96–99.5% purity)	Yes for all	2–3	HIV DNA detected by PCR. 12/34	N.D.	N.D.
Valcour et al. (111)	27	Microbead (purity Not Determined)	*n* = 26 Yes *n* = 1 No	1	HIV DNA detected by PCR. 8/27	N.D.	N.D.
Spivak et al. (112)	13	Microbead (purity not determined)	Yes for all	N/A	HIV DNA detected by PCR. LTR: 2/13, Nef: 1/10	N.D.	1.5% CD3+ cells detected
Ndhlovu et al. (113)	12	Magnetic Separation (purity not determined)	Yes for all	2–24	HIV DNA detected by PCR. 12/12	N.D.	N.D.
Hansen et al. (114)	6	FACS sorting (>98% purity)	Yes for all	9–22	HIV DNA detected by ddPCR. 6/6	N.D.	N.D.
Pasquereau et al. (115)	31	Plate adherence (purity not determined)	Yes for all	1–27	HIV DNA detected by PCR. Detection rate not stated.	N.D.	N.D.
Cattin et al. (116)	15	Microbead and FACS sorting, or FACS only (>99% purity)	Yes for all	0.3–16	HIV DNA detected by PCR Total: 4/10, Integrated: 0/10	Not detected by qVOA 0/3	N.D.

*N/A, not available; N.D, not determined; ms, multiply spliced; ddPCR, droplet digital PCR; TCR, T cell receptor; qVOA, quantitative viral outgrowth assay*.

### The Macrophage Reservoir Persists During cART-Mediated Virological Suppression

Macrophages are more permissive to HIV infection than monocytes and may be productively infected *in vivo*. Studies of individuals on effective cART are limited but have detected HIV DNA, RNA and even HIV Capsid p24 protein in tissue macrophages using multiple techniques (Table 2) (60, 80, 114, 116, 122–131). Zalar et al. detected HIV DNA in macrophages present in duodenal tissue from gut biopsies from 9 of 20 virologically suppressed HIV+ individuals, and reported CD68+ macrophages expressing p24, suggesting productive infection (122). Moreover, Ganor et al. have demonstrated replication competent HIV in urethral macrophages and measured total and integrated HIV DNA, HIV RNA and p24 in all virologically suppressed donors tested (60). Further studies have shown HIV DNA and RNA within CD68+ macrophages in brain tissue of cART-treated virologically-suppressed individuals via DNA and RNAScope (80, 127, 130), and Lamers et al. suggested that this virus is actively replicating (132), providing strong evidence for a local macrophage reservoir, which may contribute to the development of HAND.

**Table 2:** Evidence for infection and persistence of HIV in macrophages.

**Reference**	***n* =**	**Virological suppression**	**Years on cART**	**Tissue**	**Detection methodology and number positive**	**Replication competence**	**T cell specific contamination**
Zalar et al. (122)	30	*n* = 20 Yes *n* = 10 No	>5	Duodenum	HIV p24 detected in CD64+ and CD68+ in mucosal monocytes and macrophages by flow cytometry. Suppressed: 9/20, Viremic: 6/10	N.D.	CD3+ and CD4+ cells excluded
Deleage et al. (123)	9	*n* = 7 Yes *n* = 2 No	N/A	Seminal vesicles	HIV RNA and p24 detected in CD163+ macrophages in sectioned tissue via *in situ* hybridization and IHC. Detection rate not stated.	N.D.	CD3 stained but not specifically discussed
Josefsson et al. (124)	8	Yes for all	4–18	GALT	HIV DNA detected in CD3-CD4+ FACS sorted myeloid cells by PCR. GALT: 4/8	N.D.	TCR mRNA detected (not quantified)
Yukl et al. (125)	7	Yes for all	N/A	Rectum	HIV DNA and RNA detected in CD13+CD45+CD3- FACS sorted myeloid cells by qPCR. DNA: 7/7, RNA: 1/7	N.D.	<0.05% CD4+ cells detected
Cribbs et al. (126)	23	*n* = 18 Yes *n* = 5 No	N/A	Lung	HIV DNA and RNA detected in alveolar macrophages by PCR. DNA: 16/23, RNA: 8/23	N.D.	N.D.
Lamers et al. (127)	1	Yes for all	N/A	Brain	HIV DNA and RNA detected in CD163+CD68+ macrophages in sectioned tissue via RNAScope and IHC. 1/1	N.D.	N.D.
Rose et al. (128)	1	Yes for all	N/A	Cerebellum, lymph node	HIV DNA and RNA detected in CD163+CD68+ macrophages in sectioned tissue via RNAScope and IHC. 1/1	N.D.	N.D.
Hansen et al. (114)	1	Yes for all	0.5	Lung	HIV DNA and RNA detected in alveolar macrophages (>95% purity) by ddPCR. DNA: 1/1, RNA: 0/1	N.D.	N.D.
Di Napoli et al. (129)	8	*n* = 6 Yes *n* = 2 No	3	Lung	HIV DNA detected in alveolar macrophages by qPCR. 1/8	N.D.	0.3 copies of TCR DNA per myeloid cell
Tso et al. (130)	2	*n* = 1 Yes *n* = 1 No	1–9	Brain	Detection of HIV DNA and RNA in CD68+ macrophages in sectioned tissue via RNA/DNAScope and IHC. Virologically suppressed: 1/1 DNA; RNA not detected. Viremic: 1/1 DNA, 1/1 RNA.	N.D	CD4 staining: no colocalization.
Kandathil et al. (131)	8	Yes for all	1–12	Liver	HIV DNA and RNA detected in macrophages via qPCR and liver macrophage VOA. DNA: 6/8, RNA: 1/8	Viral outgrowth detected	CD3 mRNA undetectable
Ko et al. (80)	16	Yes for all	N/A	Brain	HIV DNA and RNA detected in CD68+ microglia and CD206+ perivascular macrophages by RNA/DNAScope and IHC. DNA: 16/16, RNA: 6/16	N.D.	N.D.
Ganor et al. (60)	20	Yes for all	3–22 (*n* = 16) N/A ( *n* = 4)	Urethra	Integrated HIV DNA detected in FACS sorted CD68+ macrophages via PCR. 3/3	N.D.	Not specifically assessed.
					Integrated HIV DNA, RNA and p24 detected in macrophages in sectioned tissue via FISH and IHC. DNA: 3/3, RNA: 3/3, p24: 6/6	N.D.	No colocalization of CD3 or CD4
					HIV replication from macrophage-rich urethral tissue detected by modified qVOA. 3/3	Viral outgrowth detected.	Tissue contained 25–30% CD4+ T cells
Cattin et al. (116)	8		6–18 (*n* = 7)	Colon	HIV DNA and HIV replication from FACS sorted myeloid cells detected by PCR and qVOA. DNA: 1/8, qVOA: 0/8	Viral outgrowth not detected.	CD3+ cells excluded

*N/A, Not Available; N.D, not determined; GALT, gut associated lymphoid tissue; ddPCR, droplet digital PCR; IHC, immunohistochemistry; FISH, fluorescence in situ hybridization; TCR, T cell receptor; qVOA, quantitative viral outgrowth assay*.

HIV DNA and RNA has also been detected in alveolar macrophages obtained from bronchoalveolar lavage (BAL) of virologically suppressed individuals (126) but the methodology used was not able to distinguish infected macrophages from cells that had ingested infected T cells, nor rule out T cell contamination during sampling. Nevertheless, Cribbs et al. showed significant impairment of alveolar phagocytic function in donors with detectable as compared to undetectable proviral HIV DNA in BAL cells (126), consistent with trends found in cART-naïve HIV+ individuals. As the degree of macrophage infection is small, this functional impairment is likely due to bystander effects of persistent inflammation and/or other infected cell populations with the tissue, however, unintegrated HIV DNA and incomplete or even productive infection of macrophages may also contribute to this impairment. HIV DNA has also been detected in BAL macrophages from virologically suppressed individuals in some (133) but not other studies (129). Damouche et al. were also unable to detect HIV DNA in CD14+CD206+ adipose tissue macrophages from any of 3 virologically suppressed donors tested (134). These contrasting observations may be expected given the small size of the HIV macrophage reservoir, especially given that many of these studies are limited to very small cohorts. Nevertheless, inconsistency between studies does highlight the variability between PLWH with respect to factors related to both HIV clinical history and host immunity, and emphasize the need for further studies to better characterize macrophage reservoirs in different contexts and tissues. Taken together, these data suggest that tissue macrophage reservoirs persist under suppressive cART in some PLWH and can be associated with impaired or dysregulated macrophage function. Few studies have assessed replication competence of the HIV detected within macrophages; Kandathil et al. has observed viral outgrowth from liver macrophages in only 1 of 8 donors (131) whilst Ganor et al. demonstrated replication competent DNA in urethral macrophages from all 3 donors assessed (60). These data suggest that replication competent DNA can persist during suppressive cART, but more work is required to determine the extent of the replication competent reservoir, and in which macrophage populations it can exist.

## Monocyte/Macrophage Involvement in Animal Models of HIV Infection

### Non-human Primate SIV Infection Models

Given the difficulties in obtaining tissue resident cells from humans, animal models such as the SIV-infected macaque non-human primate (NHP) model has been invaluable for studying HIV replication dynamics in macrophages. Similar to HIV infection in humans, SIV targets macrophages in addition to CD4+ T cells (135, 136) and exhibits a similar distribution within blood and tissues. Rhesus macaques are often used in reservoir and cure studies as infection is associated with sustained viral loads, progressive CD4+ T cell depletion and chronic immune activation (137), and viremia can be controlled but not eliminated by cART, leading to viral rebound if cART is interrupted (138). These similarities to HIV disease have made the SIV-macaque model extremely useful for studying the early establishment of HIV infection and reservoir dynamics in the setting of virologic suppression with cART due to access to repetitive blood sampling and ready access to appropriate tissue samples. This model has been used to demonstrate the persistence of the HIV monocyte/macrophage reservoir under cART, using modified macrophage-qVOAs to detect replication competent virus in myeloid cells from blood, brain, BAL, lungs and spleen of virologically suppressed subjects (139–141). DNA and RNAScope technology has also been leveraged to specifically detect SIV infected macrophages *in situ* and distinguish genuine macrophage infection from phagocytosis of infected T cells (79). Using DNAScope, Di Napoli et al. observed SIV DNA in splenic macrophages of macaques receiving cART for at least 5 months with undetectable viral load; however, they were unable to detect replication-competent virus from this reservoir and were also unable to detect SIV DNA in BAL macrophages (129). This may explain inconsistent detection of myeloid-associated HIV in HIV+ individuals on cART and suggests the myeloid reservoir is likely not only small and stochastic, but may also be influenced by viral and host response factors that we are yet to fully understand.

The SIV macaque model has also been used to study disease pathologies such as SIV encephalitis (SIVE). Several SIVE NHP models have been developed [reviewed in (142)], and are often derived via infection of macaques with neurotropic- and macrophage-tropic SIV strains, complemented in some studies with T cell depletion (142). These infection models lead to a higher incidence of SIVE and more rapid onset of pathogenesis compared to the slow progression of HAND observed in HIV+ individuals on suppressive cART, but they have been used to emphasize the association of SIVE with macrophage-tropic viruses, increased monocyte turnover (143, 144) and infiltration and accumulation of inflammatory macrophages into the brain (145, 146). Currently, SIVE models combined with cART are in development which may better reflect the persistence of the milder HAND observed in HIV+ individuals on suppressive cART and represent a promising new avenue for investigating the specific contribution of infected and bystander monocytes and macrophages to neuropathology.

Monocyte/macrophage targeted therapeutics have also been evaluated in the SIV model as an approach to minimize neurodegenerative disorders and cardiovascular disease. Campbell et al. investigated the effect of anti-α4 integrin blocking antibody (natalizumab), which prevents monocyte and lymphocyte trafficking into the brain and gut in SIV infected macaques. Early administration during acute infection blocked CNS infection and macrophage accumulation, and administration of the antibody during chronic infection (after established neuronal damage and macrophage accumulation) led to stabilization of neuronal injury (147), which strongly supports the critical role of infected monocytes in seeding SIV infection within the brain and the importance of myeloid cell trafficking and accumulation in the development of neurodegenerative disorders during SIV infection. Moreover, natalizumab blocks monocyte/macrophage trafficking to heart tissues which was associated with decreased cardiac fibrosis, inflammation, and cardiomyocyte degeneration (148). These findings are consistent with the observation of HIV-infected macrophages in atherosclerotic plaques (149). The correlation of monocyte and macrophage trafficking with neurodegenerative disorders and cardiovascular disease suggests the potential for pharmacological treatment of these persistent comorbidities found in HIV+ individuals on cART.

### Mouse Models of HIV Infection

Humanized mouse models have also allowed targeted studies of the monocyte/macrophage reservoir alone, or in combination with T cells, and its potential contribution in the context of cART suppression or disease states like HIV-associated encephalitis (150). Arainga et al. have demonstrated that mice transplanted with human hematopoietic stem cells (huHSC) can sustain HIV infection and respond well to cART with substantial reductions in detectable viral DNA and RNA, but similar to the response of humans, cART does not eliminate HIV reservoirs (151). Humanized BLT mice are readily infected with HIV and were validated to have detectable HIV DNA and RNA in both T cells and macrophages in the absence of cART and reduced and undetectable reservoirs, respectively, in the presence of cART (152). Honeycutt et al. have developed a humanized myeloid-only mouse (MoM) by transplanting hematopoietic stem cells into NOD/SCID mice which are unable to support human lymphocyte development. Using this novel model, they were able to show that the monocyte/macrophage reservoir can sustain infection independently of CD4+ T cells (152) and that viremia is undetectable in cART treated mice compared to cART-naïve mice (153). Upon discontinuation of cART, 3 of 9 mice examined had detectable viral rebound within 7 weeks post-treatment interruption. The presence of rebound viremia correlated with a higher viral load prior to cART initiation (153). These data demonstrate that the HIV monocyte/macrophage reservoir potentially remains a source of reactivatable virus even in a setting of suppressive cART. This MoM study is limited by a short antiretroviral treatment duration (5 weeks) and follow-up period after cART interruption (7 weeks). Also, it may not recapitulate conditions found *in vivo* in humans since human macrophage turnover in this model was estimated to be 1.05 days, which is far shorter than the estimated half-lives of normal tissue and MDM, and T cell interactions may be necessary for the persistence of the HIV macrophage reservoir. Nevertheless, data from these models are consistent with persistence and relevance of this reservoir, and are noteworthy due to limited *in vivo* data from people living with HIV.

In the MoM model, HIV infected mice have a greater accumulation of human macrophages in the brain compared to non-infected mice. HIV infection of human macrophages resident in the brain of these mice was demonstrated by immunohistochemistry for HIV capsid protein p24 and by detection of HIV RNA using qRT-PCR on RNA from isolated macrophages (152). This underscores the role of HIV-infected myeloid cell infiltration and accumulation to establish HIV reservoirs within the brain. This model has not yet been used to evaluate monocyte and macrophage infection in the context of the CNS and neuropathology but would be a highly relevant and interesting avenue for future studies.

Other mouse models have been used to evaluate the contribution of the monocyte/macrophage reservoir to HIV associated pathologies, specifically HIVE. In early studies, intracranial injection of HIV-infected human macrophages or microglia into SCID mice were used to produce a SCID-HIVE model (154) which effectively recapitulates some of the neuropathology of HIV encephalitis in humans (astrogliosis, multinuclear giant cells, and monocyte migration), and does so on an accelerated timeline compatible with the short lifespans of mice. This methodology, however, results in unavoidable confounding factors like trauma at the injection site and xenoreactivity. Attempts at using transgenic mice and humanized mouse models have had limited success in mimicking neuropathology [reviewed in greater depth in (155) and (156)]. Humanized mouse models are attractive as a model for HAND as infection in the brain can be established via a systemic route.

Mouse models have also been developed to measure HIV/SIV replication from infected human and NHP cell samples. Mice are not naturally susceptible to HIV infection; however, they can be engineered to host *in vivo* modified qVOA systems allowing for long-term viral outgrowth detection to better capture reactivation of the latent reservoir. These systems involve the xenografting of cells from HIV+ individuals into immune-modified mice and detecting virus in plasma following an incubation period to detect low frequency reactivation of latent proviruses. Two types of mouse-based qVOAs, reviewed by Schmitt and Akkina (157), have been developed: the mVOA which uses immunodeficient NOD *scid gamma* NSG mice, and the huVOA, which uses humanized Hu-HSC or BLT mice which have a reconstituted human immune system through injection of human hemopoietic stem cells (and in BLT mice, implantation of fetal liver and thymic tissue) into irradiated NSG mice. The mVOA, developed by Metcalf Pate et al. involves injecting immunodeficient NSG mice with large numbers of PBMC or CD4+ T cells, combined with antibody mediated CD8 depletion and, in some cases, CD3 activation (158). This mVOA technique has successfully detected reactivation of latent virus in PBMC derived from an elite controller with a negative *in vitro* qVOA result (158) and from other negative qVOA samples (159), but is limited by variable engraftment rates and rapid onset of graft vs. host (GvH) responses. The use of humanized mice overcomes GvH and provides a larger range of humanized target cells. HuVOAs have been validated and demonstrated to be more sensitive than qVOA (160). Both the mVOA and HuVOA are useful, ultrasensitive systems for detection of low frequencies of HIV-infected cells but have not yet been used to detect HIV in purified monocytes. These techniques may be used to evaluate future cure strategies and attempts.

Conclusions derived from the use of these mouse models are constrained by the short natural lifespan of mice which precludes studies of age-related inflammatory comorbidities found in humans with long-term cART suppression. Nevertheless, development of humanized mice susceptible to HIV infection are a valuable resource that can be used for accelerated models of disease, for studying HIV persistence and infection dynamics, and detection of low frequency infection. These mouse models are thus useful tools given the scarcity and barriers to access of human samples and their capacity to recapitulate aspects of disease pathology consistent with those found in PLWH.

## Relevance of the Monocyte/Macrophage Reservoir for HIV Cure Strategies

The myeloid HIV reservoir may be clinically relevant as it is potentially long-lived, relatively resistant to the cytopathic effects of HIV infection (161) and resistant to CTL-mediated killing (162). Moreover, being widely dispersed throughout the body, they can inhabit sanctuary sites and tissue reservoirs such as the brain and lymph tissue which may experience reduced penetrance of antiretroviral drugs (163–165). Macrophages are also intrinsically resistant to some antiretroviral drugs [reviewed in (166)]; protease inhibitors saquinavir and ritonavir showed ~2–10 fold lower activity in chronically-infected macrophages compared to chronically-infected lymphocytes (167). Moreover, *in vitro* cultures treated with antiretrovirals demonstrated that concentrations of nucleoside analogs are 5–140 fold lower in macrophages than in lymphocytes, and their antiviral activity was significantly decreased when combined with M-CSF stimulation (an M2 polarizing factor) (168). These characteristics, combined with the persistence of HIV within monocytes and macrophages, and the observation of a non-T cell source of rebound viremia in some PLWH (169), suggest the existence of a replication competent, and clinically relevant monocyte/macrophage HIV reservoir.

Current cure research is focused on T cells, the primary HIV reservoir. Cure strategies are varied and include CRISPR based gene therapy, vaccines to boost anti-HIV immune response, broadly neutralizing antibody immunotherapy approaches to cure, and the “shock and kill” strategy, in which the latent reservoir is targeted by reversing latency under continued suppressive cART and triggering cell death (170). This strategy is still in development and has seen limited success (171–178). If monocytes/macrophages are considered a legitimate reservoir which persists under cART, the effect of latency reversing interventions should be investigated in these cells. The observation that latency reversing agents (LRAs) can reactivate latently infected macrophages in the CNS leading to increased immune activation and inflammatory responses in a SIV model (179) demonstrate the potential risks of LRAs potentiating macrophage-mediated pathologies such as HAND. Moreover, CTL mediated killing activity against HIV-infected macrophages is not only ineffective but leads to increased inflammation (162). Thus, strategies to target the macrophage reservoir and minimize unintended activation may need to be investigated in parallel with T-cell centric cure strategies.

## Conclusions

The development of new animal models and HIV detection techniques coupled with greater understanding of the scope of monocyte and macrophage biology has significant implications on how HIV myeloid reservoir research is appraised. The circulation of monocytes and trafficking through tissues, including through lymph nodes which are tissue reservoirs of HIV, represents a new avenue by which monocytes can become infected and contribute to HIV persistence. Moreover, the existence of long-lived macrophage populations which can maintain a HIV reservoir via homeostatic cell division is analogous to memory T cell reservoirs and indicates the potential for tissue macrophages to be a viable HIV reservoir. With this greater understanding of how a myeloid reservoir could be seeded and maintained, coupled with mouse studies indicating that the myeloid reservoir can sustain infection and serve as the source of rebound viremia independently of T cell infection, there has been a resurgence in interest in the myeloid reservoir as a legitimate barrier to HIV cure. Current data suggests that the monocyte/macrophage reservoir is very small in PLWH and in non-human primates with cART suppression and is likely to be a minor contributor to viral rebound compared to the latent T cell reservoir. Nevertheless, the persistent monocyte/macrophage reservoir may be relevant in HIV+ individuals on cART through contribution to pathologies but also as a potential viral source which should not be ignored in attempts to cure or treat HIV.

## Author Contributions

MW wrote the article. AJ and AH conceived the topic and contributed to the manuscript.

### Conflict of Interest Statement

The authors declare that the research was conducted in the absence of any commercial or financial relationships that could be construed as a potential conflict of interest.
