# Corrigendum: The HIV Reservoir in Monocytes and Macrophages

**DOI:** 10.3389/fimmu.2019.02517

**Published:** 2019-10-22

**Authors:** Michelle E. Wong, Anthony Jaworowski, Anna C. Hearps

**Affiliations:** ^1^Central Clinical School, Monash University, Melbourne, VIC, Australia; ^2^Life Sciences Discipline, Burnet Institute, Melbourne, VIC, Australia; ^3^Chronic Inflammatory and Infectious Diseases Program, School of Health and Biomedical Sciences, Bundoora, VIC, Australia; ^4^Department of Infectious Diseases, Monash University, Melbourne, VIC, Australia

**Keywords:** HIV, monocytes/macrophages, reservoir, DNAscope, animal models

In the original article, there was an error. The individual who generated artwork for Figure 1, was not duly acknowledged.

A correction has been made to the **Acknowledgements** section.

“The authors kindly thank Mr. Hans Kek for generating the artwork for Figure 1. We gratefully acknowledge the contribution to this work of the Victorian Operational Infrastructure Support Program received by the Burnet Institute.”

The authors apologize for this error and state that this does not change the scientific conclusions of the article in any way. The original article has been updated.

